# Treatment of Dysentery

**Published:** 1883-03

**Authors:** 


TREATMENT OF DYSENTERY.

Mr. F. Rawle, M.R.C.S., observes that, at the present time, when dysentery is very prevalent, especially amongst those who have returned from the Egyptian war, any suggestion that may mitigate the suffering of so fatal a malady will be hailed with gratitude. The plan he has used with most success is the following: First, having placed the patient between warm blankets, a pint and a half of warm water, at a temperature of 90° Fahr. is injected. This is seldom retained longer than a few minutes, but is pronounced very grateful to the patient. When the water has soothed the mucous membrane of the colon and rectum, and brought away any *effete* matter, two ounces, by measure, of the following enema is administered with a gum-elastic bottle. ℞. Quinine sulphate, ten grains; compound tincture of camphor, four drachms; decoctum amyli to two ounces. Mix, and when about milk-warm, inject, which is generally retained; but, if ejected, it may be repeated after an hour or two. This has been found of great service, and very grateful to the patient; the effect is like magic. If griping pains be felt over the region of the epigastrium, half-drachm doses of chlorodyne, in some aromatic water, mint, carraway, or aniseed should be given. The diet, of course, should be of the most soothing kind; jellies, isinglass, linseed, toast and barley water *ad libitum*. Ipecacuhana appears of little service, and Mr. Rawle has discarded it from his treatment. Warm turpentine stupes on warm flannels, over the hypogastrium prove very beneficial.—*British Medical Journal.*

